# A New Narcotic

**Published:** 1879-11

**Authors:** 


### A NEW NARCOTIC.

Jamaica Dogwood, piscidia erythrina, is recommended in the *Pharmaceutical Journal* as a powerful narcotic, capable of producing sleep and relieving pain in an extraordinary manner. It has been used as an anodyne in toothache, curing the pain when introduced upon a dossil of cotton into the carious tooth. In Brazil it has an established reputation as a nervous sedative. Its action seems to be over the nerve-centers; it causes sleep without producing the cerebral hyperæmia which succeeds opium and the active principles extracted therefrom. The sleep is tranquil and refreshing; it soothes bronchial cough and moderates the paroxysm of asthma and nervous coughs. It has been used with success in chronic hepatitis and obstructions of the liver.

The idiosyncrasies encountered in many cases in regard to the action of opium and its alkaloids, compel the profession to seek an anodyne and hypnotic in other agents. We think this remedy worthy of a trial. The fluid extract is used in doses of five drops.

